# The impact of pharmacist early active consultation (PEAC) on multidrug resistance organism treatment outcomes: A prospective historically controlled study

**DOI:** 10.3389/fphar.2023.1128219

**Published:** 2023-03-02

**Authors:** Qian Du, Xin Xi, Jie Dong, Tongyan Zhang, Dongxuan Li, Yuzhu Dong, Wenjun Li, Guili Huang, Jun Zhu, Hailong Ran, Jinghui Gou, Cheng Chen, Zhanfeng Bai, Qinglong Liu, Wei Yao, Lei Zhang, Yutian Bi, Songqing Liu

**Affiliations:** ^1^ Department of Pharmacy, The Third Affiliated Hospital of Chongqing Medical University, Chongqing, China; ^2^ Infectious Disease Department, Second Affiliated Hospital of Tianjin University of Traditional Chinese Medicine, Tianjin, China; ^3^ College of Pharmacy, Chongqing Medical University, Chongqing, China; ^4^ Department of Respiratory Medicine, The Third Affiliated Hospital of Chongqing Medical University, Chongqing, China; ^5^ Department of Intensive Care Unit, The Third Affiliated Hospital of Chongqing Medical University, Chongqing, China; ^6^ Department of Medical Administration, The Third Affiliated Hospital of Chongqing Medical University, Chongqing, China

**Keywords:** clinical pharmacists, infective disease, consultation, multidrug-resistance organism, early intervention

## Abstract

**Background and aim:** Infectious disease (ID) consultation can improve multidrug-resistant organism (MDRO) treatment outcomes. However, the impact of clinical pharmacists’ ID consultation on MDRO therapy, especially early initiation, has not been reported. In this study, we try to explore the impact of the pharmacist early active consultation (PEAC) on MDRO patient management.

**Methods:** We conducted a prospective historical controlled study based on PEAC in MDRO patients. The retrospective control group was patients hospitalized 18 months before the PEAC initiation, and the prospective PEAC group was patients hospitalized 18 months after the PEAC initiation. Primary endpoint was 30-day all-cause mortality. Secondary outcomes were MDRO clinical outcome, duration of antibiotic use, length of stay, antibiotic consumption and antibiotic costs. Further subgroup analysis of secondary outcomes was performed by the condition at admission, MDRO pathogenicity and MDRO clinical outcome.

**Results:** 188 MDRO patients were included. After adjusting for potential predictors, PEAC reduced the 30-day all-cause mortality by 70% (HR 0.30, 95% CI 0.09–0.96, *p* = 0.042). PEAC group had clinical improvement than control group (89.47% vs. 65.59%, *p* < 0.001), especially in patients with non-severe clinical conditions at admission (98.41% vs. 70.18%, *p* < 0.001). However, no significant differences were found between groups in length of stay, antibiotics consumption, and antibiotics costs.

**Conclusion:** Early active pharmacy ID consultation can reduce 30-day all-cause mortality and improve clinical outcomes in MDRO patients.

## 1 Indroduction

Bacterial drug resistance has become an urgent public health threat. The spread of multidrug-resistant organisms (MDRO) has resulted in a continued increase in morbidity, reduced treatment options, increased mortality, prolonged hospital stays, and increased costs (Weiner et al., 2016; Burnham et al., 2018a; Majumder et al., 2020; Chen et al., 2021; Zhen et al., 2021; Zhou et al., 2021). The MDRO carrier status must be distinguished from colonization and infection. Colonization occurs in more cases. However, MDRO is often overtreated as it is challenging to determine colonization from infection accurately. Meanwhile, effective treatment of MDRO infections is often delayed (Blot et al., 2005; MacVane et al., 2014; Friedman et al., 2016). Although anti-infective disease (ID) consultation can improve the efficacy of drug-resistant bacteria treatment and reduce mortality, it is not timely because it is a passive process. ID consultation is only requested when the treating physician thinks it is necessary, and treatment failure is often experienced (Tissot et al., 2014; Farmakiotis et al., 2015). Therefore, more effective management strategies and optimal drug therapies are required to initiate timely for MDRO patients.

Increasing studies have shown that clinical pharmacists can play a significant role as essential members of the antimicrobial stewardship (AMS) treatment team. These include optimizing anti-infective treatment strategies and reducing treatment costs through pharmacy consultations and other pharmacy service models (Beach et al., 2017; Lin et al., 2019; Zhang et al., 2019; Zhang et al., 2020). In the first half of 2018, clinical pharmacists at our institution found that the number of MDRO patients increased gradually. At the same time, clinical pharmacists found that there was still room for improvement of MDRO treatment management in pharmaceutical consultation. In one hand, inexperienced physicians may select inappropriate anti-infection scheme without anti-infection expert guidance. On the other hand, traditional pharmacy consultation is usually initiated by doctors, which can cause delays for patients receiving the opinion of the pharmaceutical consultation.

In order to optimize the MDRO treatment management, we established a “Pharmacist Early Active Consultation (PEAC)" pharmacy service for MDRO patient at our hospital. PEAC was a spontaneous active pharmacy consultation conducted by clinical pharmacists for all MDRO drug susceptibility reports within 24 h, aiming at providing optimal and timely anti-infective treatment for MDRO patients. This study explored the impact of the PEAC pharmacy service on the treatment effect, hospitalization expenses, and antimicrobial use of MDRO patients.

## 2 Materials and Methods

The study was conducted at the Third Affiliated Hospital of Chongqing Medical University, a comprehensive tertiary teaching hospital located in Chongqing, China, which has 1,350 beds with an average of 40,000 inpatients per year. Our hospital attached great importance to the practice of AMS, and performed AMS activities according to the requirements of the National Health Commission of China. Clinical pharmacists in our hospital play an essential role in AMS activities including setting up an antibacterial drug management working group, carrying out graded management and authorization of antibacterial drugs, restricting the use of high-grade antibiotics by conducting prescription comments, and providing pharmaceutical consultation for physicians and patients.

### 2.1 The pharmacists’ early active consultation pharmacy service

On 1 July 2018, clinical pharmacists in our hospital began to implement PEAC pharmacy services for hospitalized MDRO patients, and all clinical pharmacists undertaking this work completed the training required by the National Health Commission of China after graduation. In infectious patient management, physicians usually write orders to carry out microbiological culture for guiding anti–infective therapy. Once the culture result indicates MDRO, the microbiology laboratory will issue the anti-infective drug susceptibility profiling report in the microbiology information system. At a fixed time in each working day, the clinical pharmacists will review all the newly released MDRO reports by logging into microbiology information system. After reviewing the MDRO report, active ID consultation will be accomplished within 24 h by clinical pharmacists. Active ID consultation includes 1) determining the pathogenicity of MDRO to ensure antibiotics use indications, 2) interpreting the results of MDRO reports to physician and patient, 3) selecting appropriate antibiotics according to drug pharmacokinetics and pharmacodynamics characteristics, 4) adjusting and optimizing drug doses based on the patient’s response, 5) implementing targeted antimicrobial therapies as soon as possible, and 5) monitoring adverse drug reactions (ADR) and proposing ADR interventions. The pharmacists conducted the first round of consultation with the physicians after obtaining the MDRO report for each patient. They performed follow-up consultations based on the patient’s response to antibiotic treatment during the treatment course. All consultation records were recorded in the Hospital Information System (HIS).

### 2.2 Study design and study population

The study was a pre-post intervention study based on the time of PEAC implementation. The results were compared in the pre-intervention period (non-PEAC group, 1 January 2017, and 30 June 2018) and the post-intervention period (PEAC group, 1 July 2018, to 31 December 2019). All MDRO carriers in these two periods were eligible for inclusion in the study. Exclusion criteria were patients 1) under 18 years of age, 2) who were discharged, died, or started palliative care within three days of receiving the MDRO report or before the MDRO report was available, 3) patients in the non-PEAC group but received pharmacy consultation within 24 h, 4) patients in the PEAC group but did not receive pharmacy consultation within 24 h due to clinical pharmacists missing the MDRO reports, and 5) who were hospitalized in the rehabilitation department as these patients would have extended hospital stays.

### 2.3 Ethics

This study was approved by the medical ethics committee of the Third Affiliated Hospital of Chongqing Medical University (No. 2018-16).

### 2.4 Definitions related to MDRO

MDRO in our study included carbapenem-resistant *Acinetobacter* baumannii (CRAB), carbapenem-resistant *Pseudomonas aeruginosa* (CRPA), carbapenem-resistant Enterobacterales (CRE), and methicillin-resistant *Staphylococcus aureus* (MRSA). These bacteria are on the priority list released by the WHO in 2017 to encourage research and development of effective drugs against these pathogens. In the study, the VITEK-compact automatic microbiological analysis system (France BioMérieux) was used to identify bacterial species and test antimicrobial susceptibility. All microbiological test methods were consistent with CLSI guidelines for the corresponding year, and antimicrobial susceptibility was determined using the CLSI breakpoints.

MDRO was classified into infection, colonization, and contamination categories. Categorization was based on the patient’s infection-related clinical symptoms, the source of specimens, the common pathogens at the infection site, and the coverage of antimicrobial treatment at the time of the MDRO report. If a patient had no infection symptoms or symptoms improved significantly, especially when the bacterial sample was a sputum specimen, it would be judged as colonization. Contamination was if the bacteria only appeared once in multiple cultures and was inconsistent with the infection symptoms. MDRO-caused infection was highly suspected if the patient’s infection symptoms persisted or aggravated while on an antibiotic regimen that could cover common pathogens of nosocomial infection, especially when bacterial specimens were sterile, such as blood or cerebrospinal fluid. For difficult-to-judge cases, cases were jointly judged by three hospital infectious disease experts to reach a consensus.

### 2.5 Outcome assessment

The primary endpoint of this study was 30-day all-cause mortality, which refers to patients who died, from any cause, within 30 days after receiving the MDRO report. Secondary endpoints were infection treatment outcome, length of hospital stay (LOS), duration and consumption of antibiotics after receiving MDRO reports, and the cost of antibiotics. We performed subgroup analyses of secondary outcomes in terms of the pathogenicity of the MDRO, the severity of the condition admitted, and the infection treatment outcome.

The outcome of infection treatment judgement methods referred to other two published studies (Lin et al., 2019; Zhang et al., 2019), which classified as improved, unchanged, or deteriorated. The improvement was that the patients had improved infection symptoms, infection indicators (white blood cell count, neutrophil ratio, procalcitonin), imaging, and normalized body temperature. Deterioration was defined as worsening clinical infection indicators. Unchanged meant no significant change in clinical infection indicators.

Antimicrobial consumption was calculated by calculating each patient’s defined daily doses (DDD) and summing all DDDs per person during hospital stays or after the MDRO reports were obtained. The use of DDDs allows one to compare the consistency of antimicrobial consumption between different drugs. DDD in adults was obtained from the WHO Anatomical Therapeutic Chemical (ATC) Classification System. DDD units are expressed in grams. We converted each patient’s grams of antimicrobials to a sum of DDD to obtain the consumption data for a single patient (Chen et al., 2018). $\text{DDDs}=\sum \left(\text{drug}\;\text{specification}\times \text{amount}/\text{defined}\;\text{daily}\;\text{dose}\right)$ .

The severity of the condition admitted was measured by a score of the United Kingdom National Early Warning Score 2 (NEWS2) (Smith et al., 2019). NEWS2 score is a disease score and mortality severity estimation tool that has been validated and proven to be helpful in the prehospital setting and is commonly used internationally (Silcock et al., 2015; Mellhammar et al., 2019; Pirneskoski et al., 2019; Smith et al., 2019). A NEWS2 score ≥7 is the urgent response threshold, which indicates that the patient is in a serious condition and requires acute treatment at admission. A score of <7 indicates a mild to medium condition at admission.

### 2.6 Data collection

Data collected included patient demographics, comorbidities, the NEWS2 recorded within 24 h after admission, microbiological data, antimicrobial consumption and cost, the total hospitalization cost, and clinical outcomes. All data were obtained from the HIS.

### 2.7 Statistical analysis

Fisher’s exact test was used to identify differences between categorical variables. Student’s t-test or Mann-Whitney *U*-test were used when appropriate for continuous variables. Categorical data are expressed as frequencies. Continuous variables are expressed as mean and standard deviation or median and interquartile range. Kaplan-Meier survival analysis was performed using a log-rank test to assess the effect of PEAC on 30-day mortality between groups. In multivariate analysis, potential predictors of 30-day mortality were first evaluated in the univariate analysis and then included in the final Cox proportional hazards model when their *p*-values were ≤0.2. Cox survival analysis was performed using Wald’s test to assess the effect of predictors on 30-day mortality between groups, and the results of Cox analysis were expressed as hazard ratios (HR). All statistical tests were two-sided, with *p* < 0.05 as the significance level. Data were analyzed using R (version 4.1.0).

## 3 Results

### 3.1 Baseline characteristics

During the 36 months (18 months before and after PEAC), 280 hospitalized patients had MDRO reports. Of these patients, 92 were excluded according to the exclusion criteria (10 under 18 years of age; 46 were discharged, died, or started palliative care within three days after receiving the MDRO report or before the MDRO report was available; 26 were hospitalized in the rehabilitation department; 7 were in the non-PEAC group but received pharmacy consultation within 24 h; 3 were in the PEAC group but did not receive pharmacy consultation within 24 h). Thus, 188 patients were analyzed, including 93 (49%) patients in the non-PEAC group and 95 (51%) in the PEAC group. The baseline and MDRO clinical characteristics in both groups are described in Table 1.

**TABLE 1:** Characteristics of the patients at the baseline.

Characteristic	All patients (*N* = 188)	Non-PEAC *(n* = 93)	PEAC (*n* = 95)	*p-*value
Age, mean, y (SD)	59.66 ± 17.02	63.09 ± 15.72	56.31 ± 17.64	**0.006** **
Male	133 (70.74)	68 (73.11)	65 (68.42)	0.523
Inpatient Days, median, d (IQR)	26.50 (15.75–55.00)	26.00 (15.00–56.50)	27.00 (16.00–54.00)	0.666
Hospitalization to MDRO, mean, d (IQR)	9.00 (4.00–21.00)	9.00 (4.50–22.00)	9.00 (4.00–20.00)	0.574
Hospital-acquired infection	152 (80.85)	79 (84.95)	73 (76.84)	0.195
ICU-acquired MDRO	30 (15.96)	13 (13.98)	17 (17.89)	0.552
NEWS2 at admission				
≥7	68 (36.17)	36 (38.71)	32 (33.68)	0.544
<7	120 (63.83)	57 (61.29)	63 (66.32)	0.544
Comorbidity				
Cardiovascular disease	7 (3.72)	3 (3.23)	4 (4.21)	>0.999
Cerebrovascular diseases	24 (12.76)	12 (12.90)	12 (12.63)	>0.999
Respiratory diseases	75 (39.89)	50 (53.76)	25 (26.32)	**<0.001** **
Traumatic diseases	23 (12.23)	8 (8.60)	15 (15.79)	0.181
Genito-urinary diseases	7 (3.72)	3 (3.23)	4 (4.21)	>0.999
Digestive diseases	17 (9.04)	6 (6.45)	11 (11.58)	0.310
Other diseases	35 (18.61)	11 (11.83)	24 (25.26)	**0.024** **
Infection site				
Respiratory	137 (72.87)	80 (86.02)	57 (60.00)	**<0.001** **
Urinary tract	25 (13.3)	12 (12.9)	13 (13.68)	>0.999
Bloodstream	15 (7.98)	10 (10.75)	5 (5.26)	0.188
Intravascular catheter	3 (1.60)	0 (0.00)	3 (3.16)	0.246
Intracranial	11 (5.85)	7 (7.53)	4 (4.21)	0.369
Intra-abdominal	14 (7.45)	6 (6.45)	8 (8.42)	0.782
Skin and soft tissue	15 (7.98)	5 (5.38)	10 (10.53)	0.282
Surgical site	5 (2.66)	0 (0.00)	5 (5.26)	0.059
Other site	10 (5.32)	2 (2.15)	8 (8.42)	0.100
Microbiological specimens				
Sputum or bronchoalveolar lavage fluid	133 (70.74)	76 (81.72)	57 (60.00)	**0.001** **
Urine	11 (5.85)	3 (3.23)	8 (8.42)	0.213
Blood	5 (2.66)	2 (2.15)	3 (3.16)	>0.999
Catheter tips	13 (6.91)	8 (8.60)	5 (5.26)	0.403
Cerebrospinal fluid	5 (2.66)	2 (2.15)	3 (3.16)	>0.999
Fluid drainage	15 (7.98)	5 (5.38)	10 (10.53)	0.282
Others	29 (15.43)	7 (7.53)	22 (23.15)	**0.003** **
MDRO				
CRAB	85 (45.21)	48 (51.61)	37 (38.95)	0.107
CRPA	52 (27.66)	30 (32.26)	22 (23.16)	0.193
MRSA	43 (22.87)	11 (11.83)	32 (33.68)	**<0.001** **
CRE	8 (4.26)	4 (4.30)	4 (4.21)	>0.999
Pathogenicity				
Colonization	88 (46.81)	46 (49.46)	42 (44.21)	0.559
Infection	93 (49.47)	43 (46.24)	50 (52.63)	0.386
Contamination	7 (3.72)	4 (4.30)	3 (3.16)	0.719

** Significant at *p* ≤ 0.05.

Data are presented as No. of patients (%) unless specified otherwise. A patient may have multiple infection site and microbiological specimens. NEWS2, National Early Warning Score 2 (severity of illness score and mortality estimation tool). NEWS2 ≥ 7 means the patient’s clinical condition was serious at admitting and demanding emergency treatment; <7 represents the mild to medium condition at admission to the hospital. PEAC, pharmacist early active consultation; IQR, interquartile range; SD, standard deviation; MDRO, multi-drug resistant organisms; CRAB, carbapenem-resistant *Acinetobacter baumannii*; CRPA, carbapenem-resistant *Pseudomonas aeruginosa*; MASA, methicillin-resistant *Staphylococcus aureus*; CRE, carbapenem-resistant Enterobacteriaceae.

There were significant differences in age, comorbidity of respiratory or other diseases, infection site of the respiratory system, specimens of sputum or bronchoalveolar lavage fluid, and MRSA infection. A total of 93 (49.47%) patients had infections caused by MDRD, 50 (52.63%) in the PEAC group and 43 (46.24%) in the non-PEAC group.

### 3.2 Pharmacist interventions in the PEAC group

A total of 386 pharmacy consultation records were conducted on patients in the PEAC group, an average of 4.1 per patient. Pharmacists proposed to change antibiotic regimens in 37 (38.95%) patients after receiving MDRO susceptibility reports. In the 50 patients with MDRO-caused infections, 32 (64%) were recommended to change antibiotic regimens. For the 40 patients with a colonized or contaminated MDRO, the clinical pharmacist advised doctors to maintain the current treatment regimen and medication monitoring points to avoid overtreatment and medication damage. Moreover, due to inappropriate treatment, a change in treatment strategy was recommended in 5 patients with a colonized or contaminated MDRO on the pharmacists’ advice. Physicians accepted the pharmacists’ recommendations for 88 patients (92.63%), partially accepted for 4 (4.21%) patients, and rejected for three patients (3.16%).

### 3.3 30-Day all-cause mortality

**FIGURE 1:**
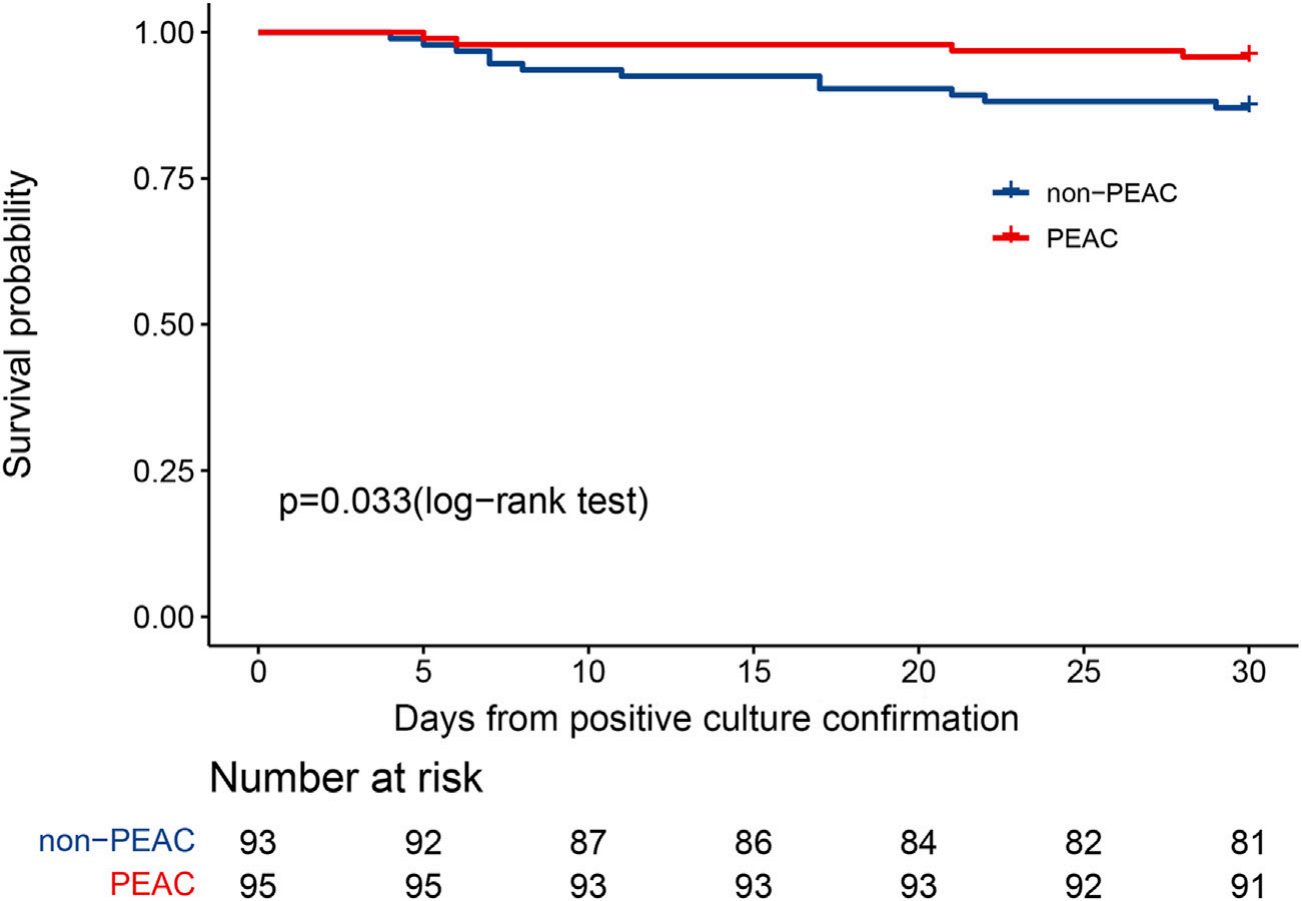
Kaplan–Meier curve illustrating the effect of pharmacist early active consultation (PEAC) vs. non-PEAC on 30-day all-cause mortality after positive MDRO culture.

**FIGURE 2:**
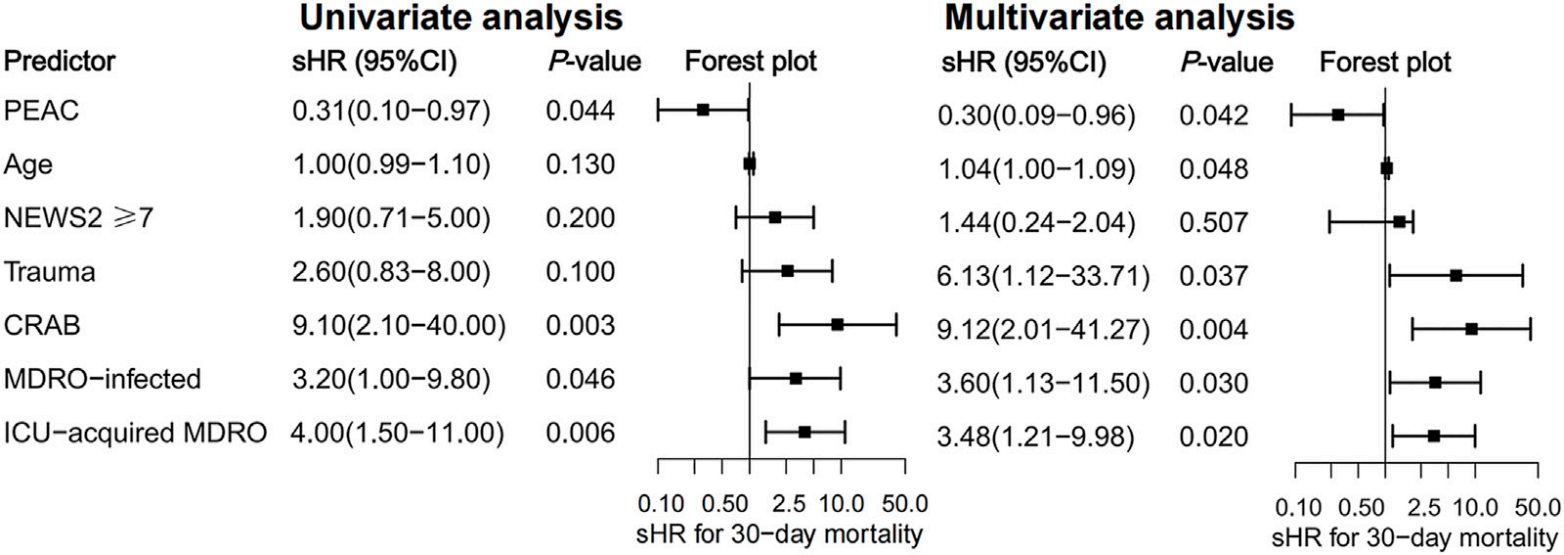
Univariate and multivariate analyses represent the relative risks (HR) of potential predictors for 30-day all-cause mortality and 95% confidence intervals (95% CI).

All patients had 30-day follow-up data. At 30 days, there were 12 deaths (12.90%) in the non-PEAC group compared to 4 deaths (4.21%) in the PEAC group. The cumulative probability of death at 30 days increased significantly in the non-PEAC group (*p* = 0.033), as shown in Figure 1. At the same time, Cox’s proportional assumption did not reject the PEAC variable. In the final multivariate COX model, the factors significantly associated with an increase in mortality were non-PEAC, older age, traumatic illness, positive CRAB culture, MDRO-caused infection, and MDRO acquired in the intensive care unit (ICU) (Figure 2). After adjusting for these predictors in the multivariate model, PEAC reduced the 30-day risk of all-cause mortality by 70% (HR 0.25, 95% confidence interval CI, 0.09–0.96; *p* = 0.042).

### 3.4 Comparisons of infection treatment outcome

Compared to the non-PEAC group, more patients in the PEAC group had an improvement in infection (*p* < 0.001), regardless of whether they had MDRO-caused infection (*p* = 0.002) or colonization (*p* = 0.015). Compared to patients admitted to the hospital with NEWS2 ≥ 7, patients with NEWS2 < 7 benefited more from PEAC, with a higher infection improvement rate (98.41% vs. 70.18%, *p* < 0.001) and a lower deterioration rate (1.59% vs. 15.79%, *p* = 0.006). However, the overall reduction in the rate of infection deterioration was not statistically significant between the PEAC and non-PEAC groups (7.37% vs. 16.13%, *p* = 0.072). Details are provided in Table 2.

**TABLE 2:** Efficacy against MDRO infections with or without PEAC.

	Number of comparisons^a^	Non-PEAC		PEAC		*p*-value
		*N* = 93		*N* = 95		
	Non-PEAC/PEAC	N	Rate (%)	N	Rate (%)	
Improvement	93/95	61	65.59	85	89.47	<**0.001** **
NEWS2 at admission						
≥7	36/32	21	58.33	23	71.88	0.312
<7	57/63	40	70.18	62	98.41	<**0.001** **
Pathogenicity						
Colonization	46/42	35	76.09	40	95.24	0.015
Infection	43/50	23	53.49	42	84.00	**0.002** **
Contamination	4/3	3	75.00	3	100.00	>0.999
Deterioration	93/95	15	16.13	7	7.37	0.072
NEWS2 at admission						
≥7	36/32	8	22.22	6	18.75	0.718
<7	57/63	7	12.28	1	1.59	**0.034** **
Pathogenicity						
Colonization	46/42	4	8.70	0	0.00	0.118
Infection	43/50	10	23.26	7	14.00	0.290
Contamination	4/3	1	25.00	0	0.00	>0.999
Unchanged	93/95	17	18.28	3	3.16	**0.001** **
NEWS2 at admission						
≥7	36/32	7	19.44	3	9.38	0.314
<7	57/63	10	17.54	0	0.00	<**0.001** **
Pathogenicity						
Colonization	46/42	7	15.22	2	4.76	0.161
Infection	43/50	10	23.26	1	2.00	**0.002** **
Contamination	4/3	0	0.00	0	0.00	>0.999

** Significant at *p* ≤ 0.05.

^a^ This column of data is used as the denominator to calculate the rate separately.

NEWS2: National Early Warning Score 2 (severity of illness score and mortality estimation tool). NEWS2 ≥ 7 means the patient’s clinical condition was serious at admitting and demanding emergency treatment; <7 represents the mild to medium condition at admission to the hospital. PEAC, pharmacist early active consultation; IQR, interquartile range.

### 3.5 Analysis of treatment duration

There were no significant differences between the two groups in total LOS (*p* = 0.666), LOS after the MDRO report (*p* = 0.349), and duration of antimicrobial course after MDRO reports (*p* = 0.911). However, total LOS (*p* = 0.040) and LOS after MDRO reports (*p* = 0.021) were markedly prolonged in patients with severe clinical conditions at admission (NEWS2 ≥ 7). The findings are summarized in Table 3.

**TABLE 3:** Treatment duration of MDRO patients with or without PEAC.

	Number of comparisons	Non-PEAC		PEAC		*p*-value
		*N* = 93		*N* = 95		
	Non-PEAC/PEAC	Median	IQR	Median	IQR	
Total LOS, days	93/95	26.00	15.00–56.00	27.00	16.00–54.00	0.666
NEWS2 at admission						
≥7	36/32	22.00	15.00–59.00	41.50	25.25–68.00	**0.040** **
<7	57/63	28.00	15.00–55.00	23.00	14.50–50.50	0.328
Pathogenicity						
Colonization	46/42	26.50	16.00–38.50	32.00	19.25–47.50	0.525
Infection	43/50	28.00	13.00–62.50	26.50	11.75–66.25	0.717
Outcome						
Improvement	61/85	23.00	15.00–41.00	28.00	17.00–54.00	0.177
Deterioration	15/7	29.00	17.00–97.50	22.00	23.00–37.00	0.640
Post-MDRO LOS, days	93/95	11.00	7.00–26.00	14.00	7.00–34.00	0.349
NEWS2 at admission						
≥7	36/32	9.50	5.75–20.00	25.50	12.00–40.75	**0.021** **
<7	57/63	14.00	7.00–26.00	12.00	7.00–27.00	0.524
Pathogenicity						
Colonization	46/42	11.00	7.00–22.75	12.50	7.25–27.00	0.485
Infection	43/50	13.00	7.50–36.50	17.00	7.25–39.75	0.373
Outcome						
Improvement	61/85	11.00	8.00–22.00	14.00	8.00–35.00	0.152
Deterioration	15/7	13.00	7.50–50.00	12.00	5.50–24.50	0.646
Post-MDRO antibiotic course, days	93/95	9.00	5.00–20.00	9.00	5.00–20.00	0.911
NEWS2 at admission						
≥7	36/32	9.00	5.00–24.50	15.00	6.00–27.50	0.392
<7	57/63	8.50	4.00–19.00	8.00	5.00–14.00	0.877
Pathogenicity						
Colonization	46/42	8.00	4.50–19.50	7.00	5.00–20.00	0.986
Infection	43/50	9.00	6.00–20.50	13.00	6.00–20.00	0.817
Outcome						
Improvement	61/85	8.00	5.00–17.50	9.00	5.25–19.25	0.361
Deterioration	15/7	9.00	6.00–27.00	12.00	5.50–24.50	0.860

** Significant at *p* ≤ 0.05.

NEWS2: National Early Warning Score 2 (severity of illness score and mortality estimation tool). NEWS2 ≥ 7 means the patient’s clinical condition was serious at admitting and demanding emergency treatment; <7 represents the mild to medium condition at admission to the hospital. PEAC, pharmacist early active consultation; LOS, length of stay; MDRO, multi-drug resistant organisms; IQR, interquartile range; MDRO, multi-drug resistant organisms.

### 3.6 Analysis of antibiotics consumption and cost

As shown in Table 4, there were no significant differences in DDDs throughout the hospital stay between the two groups of patients (*p* = 0.647). PEAC increased antibiotic DDDs after the MDRO reports, but no significant statistical differences were found (10 vs. 7.75, *p* = 0.052). In patients admitted to the hospital with severe disease (NEWS2 ≥ 7), PEAC significantly increased overall DDDs (61.81 vs. 38.59, *p* = 0.040) and DDDs after MDRO reports (30.07 vs. 10.64, *p* = 0.028). Similar trends were observed in patients with NEWS2 ≥ 7 (*p* = 0.015), who were ultimately judged to have improved infection effects (*p* = 0.019). Details are presented in Supplementary Tables 1, 2. PEAC did not significantly increase the cost of antimicrobials after the MDRO reports (*p* = 0.911), even in patients with MDRO-caused infections.

**TABLE 4:** Antibiotic consumption and cost for MDRO patients with or without PEAC.

	Number of comparisons	Non-PEAC		PEAC		*p*-value
		*N* = 93		*N* = 95		
	Non-PEAC/PEAC	Median	IQR	Median	IQR	
Total DDDs	93/95	26.50	13.27–52.63	25.20	11.71–70.77	0.647
NEWS2 at admission						
≥7	36/32	38.59	19.15–50.77	61.81	28.18–108.44	**0.040** **
<7	57/63	23.43	9.00–53.90	17.40	9.61–38.50	0.668
Pathogenicity						
Colonization	46/42	27.43	13.58–48.94	26.80	10.82–46.84	0.957
Infection	43/50	24.00	14.89–64.06	27.60	13.21–85.26	0.584
Outcome						
Improvement	61/85	23.75	13.27–49.00	25.20	11.55–68.50	0.484
Deterioration	15/7	49.25	22.51–74.73	65.96	22.67–119.24	0.597
Post-MDRO DDDs	93/95	7.75	1.77–21.64	10.00	4.38–38.83	0.052
NEWS2 at admission						
≥7	36/32	10.64	2.00–26.16	30.07	4.44–69.56	**0.028** **
<7	57/63	6.29	1.64–19.25	8.40	4.05–21.30	0.266
Pathogenicity						
Colonization	46/42	4.25	1.66–16.6	6.37	0.78–17.70	0.873
Infection	43/50	11.25	3.06–32.23	21.30	6.63–65.93	**0.046** **
Outcome						
Improvement	61/85	6.29	1.77–20.8	10.00	4.50–37.67	0.051
Deterioration	15/7	15.20	3.50–27.8	28.79	7.73–81.45	0.217
Antibiotic cost	93/95	1513.35	611.08–3306.33	1324.23	393.26–2742.5	0.373
NEWS2 at admission						
≥7	36/32	2144.03	887.15–3061.39	2468.75	1842.64–4465.14	0.151
<7	57/63	1285.79	489.47–3307.99	746.32	285.09–2002.6	0.149
Pathogenicity						
Colonization	46/42	1371.92	636.44–3375.86	1207.96	453.27–2717.01	0.437
Infection	43/50	1782.08	643.22–3295.07	1345.03	444.09–2774.26	0.641
Outcome						
Improvement	61/85	1228.30	505.64–2735.1	1204.18	382.05–2738.98	0.783
Deterioration	15/7	2987.25	1562.8–4103.16	2322.12	1727.11–9055.25	0.647

** Significant at *p* ≤ 0.05.

The cost was at the exchange rate as US $100 is approximately equal to 636.53 Chinese Renminbi (Date of conversion 9 April 2022). Antibiotic cost is the sum of expense of antibiotic drug, whatever they were used to treat MDRO or not. NEWS2: National Early Warning Score 2 (severity of illness score and mortality estimation tool). NEWS2 ≥ 7 means the patient’s clinical condition was serious at admitting and demanding emergency treatment; <7 represents the mild to medium condition at admission to the hospital. Abbreviation: PEAC, pharmacist early active consultation; DDD, defined daily dose; MDRO, multi-drug resistant organisms; IQR, interquartile range.

## 4 Discussion

This study described a timely and active pharmacy ID consultation in optimizing antibiotic drug therapies after receiving MDRO reports. The population included patients with four types of highly resistant MDRO regardless of the site of infection, MDRO-carrier status and the severity of the disease at admission. Various outcomes were assessed, including 30-day all-cause mortality.

Our study reported the pharmacist early active consultation in MDRO patients could reduce the 30-day risk of all-cause mortality by 70%. This result was of concern and impressive. There have been some reports about the positive impact on consultation for ID, yet most have focused on the role of physicians (Hamandi et al., 2014; Tissot et al., 2014; Bai et al., 2015; Farmakiotis et al., 2015; Vogel et al., 2016; Beach et al., 2017; Spec et al., 2017; Burnham et al., 2018b; Mejia-Chew et al., 2019), and a few on clinical pharmacists (Lin et al., 2019; Zhang et al., 2019). However, no studies have reported the effects of ID consultation by infection specialists on MDRO therapy. There are several reasons for the positive effects of PEAC on MDRO treatment. The first reason is that infection may occur in various organs, but the ability of specialists to diagnose and treat complex infectious diseases is inadequate, especially on the judgment of the MDRO pathogenic and the interpretation of the MDRO drug susceptibility report. Second, our hospital did not yet have the infectious disease department during the research period, which made clinical pharmacists with infection-related training backgrounds assume a considerable responsibilities of ID experts. In addition, clinical pharmacists participated in treatment on all MDRO inpatients as early as possible, even on the day of the MDRO report, and early intervention can significantly improve the prognosis of infectious diseases. Two studies mentioned the impact of early ID intervention. One found that mandatory ID consultation reduced mortality in patients with MRSA bacteremia if the consultation was completed within 48 h of the availability of MDRO reports (Tissot et al., 2014). In our study, pharmacists-initiated ID consultations earlier, within 24 h. Another study also demonstrated a positive relationship between 24 h early intervention and reduced 28-day and in-hospital mortalities in cancer patients with *Candida* glabrata (Mejia-Chew et al., 2019). Lastly, clinical pharmacists will continue to follow up with MDRO patients after the first time of PEAC, paying close attention to patient condition changes to timely adjust the treatment plan, so that the treatment can be timely adjusted and medication damage can be avoided.

However, there were no significant differences in overall hospital stay, course of anti-infective treatment and antibiotic consumption after MDRO reports, and antibiotic costs. The results demonstrated that PEAC did not cause higher costs, extended hospital stays, and duration of treatment. Our findings are consistent with a study conducted in the Netherlands in which optimizing antibiotic regimens did not significantly reduce antibiotic consumption and LOS (Sikkens et al., 2017). It should be noted that among the patients in the NEWS2 ≥ 7 groups, PEAC led to more extended hospitalization, especially in patients with improved clinical infections. However, the total consumption and cost of antibiotics and the duration of treatment did not increase. These patients were more seriously ill at admission, and infection was difficult to control during hospitalization, resulting in high mortality risk. In implementing PEAC, clinical pharmacists provided these patients with more optimized antibiotic treatment and conducted persistent follow-ups to timely adjust anti-infection treatment. Those measures can improve the treatment effect for those MDRO patients, although there is no statistical significance in increasing the therapeutic effect due to the small sample of patients. In our study, patients with NEWS score ≥7 in the PEAC group have a longer total LOS and LOS after positive MDRO culture. We speculate that this result can be interpreted as those patients will benefit from PEAC and get more opportunities to treat primary diseases, so their LOS significantly increased. Although the rate of clinical improvement did not decrease significantly in patients with NEWS2 ≥ 7 under the PEAC intervention, this could be related to the smaller number of samples. For patients with NEWS2 < 7, PEAC significantly increased the clinical improvement rate, indicating that PEAC benefited patients with relatively mild diseases more.

Our study has several limitations. First, the samples size of our study was small, which could have limited the ability to detect differences in outcome parameters in the two groups. Second, Due to the retrospective analysis nature of the non-PEAC group, the infection treatment status of some patients could not be judged accurately. Their treatment effects appeared stable if solely judged from the medical records, with no significant improvement or deterioration in symptoms and examinations related to infections. Those uncertain treatment effects may bias the analysis result in an unknown way, but it could be also a reflection of insufficient treatment. The number of “unchanged” patients was significantly lower in the PEAC group, which could partly attribute to PEAC resulted some “unchanged” patients improved under the intervene of clinical pharmacist. Third, the diagnosis and treatment of patients need the close cooperation of doctors, nurses, pharmacists and other medical personnel, and patient recovery in the study cannot separate from the joint assistance of all medical staff. Our study only showed the positive impact of the active pharmaceutical service for MDRO management provided by clinical pharmacist, but we did not assess the influence of other medical personnel’ personal ability growth over time, which may lead to biased result.

## 5 Conclusion

The timely intervention of clinical pharmacists for MDRO patients reduced 30-day all-cause mortality and improved the clinical outcomes of antibiotic treatment without increasing hospital stay, and antibiotic consumption and antibiotic cost. Our study contributes to the body of literature that demonstrates the impact of pharmacist interventions on managing patients with infectious diseases. It also shows that timely and effective intervention of proper anti-infective treatment is crucial for MDRO patients.

## Data Availability

The original contributions presented in the study are included in the article/Supplementary Material, further inquiries can be directed to the corresponding authors.
