# Supplementary material for: The impact of pharmacist early active consultation (PEAC) on multidrug resistance organism treatment outcomes: A prospective historically controlled study
Source: Front Pharmacol. 2023 Mar 2;14:1128219. doi: 10.3389/fphar.2023.1128219 (PMC10017476; doi:10.3389/fphar.2023.1128219)
Supplement: Supplementary file 1 [file DataSheet1.docx]

Supplementary Material

The Impact of Pharmacy Early Active Consultation (PEAC) on Multidrug Resistance Organism Treatment Outcomes: A Prospective Historically Controlled Study

Qian Du, Xin Xi, Jie Dong, Tongyan Zhang, Dongxuan Li, Yuzhu Dong, Wenjun Li, Guili Huang, Jun Zhu, Hailong Ran, Jinghui Gou, Cheng Chen, Zhanfeng Bai, Qinglong Liu, Wei Yao, Lei Zhang, Yutian Bi^*^, Songqing Liu^*^

*** Correspondence:**

Yutian Bi: biyutian2@163.com

Songqing Liu: liusq@hospital.cqmu.edu.cn

**Supplementary Table 1. Efficacy Evaluation between Different Disease Severity^a^ at Admission**

|  | NEWS 2 ≥7 | | | NEWS 2 <7 | | |
| --- | --- | --- | --- | --- | --- | --- |
|  | non-PEAC  N=36 | PEAC  N=32 | *P-*Value | non-PEAC  N=57 | PEAC  N=63 | *P-*Value |
| **Infection treatment effect (n, %)** |  |  |  |  |  |  |
| Improvement | 21 (58.33) | 23 (71.88) | 0.312 | 40 (70.88) | 62 (98.41) | <0.001 |
| Deterioration | 8 (22.22) | 6 (18.75) | 0.772 | 9 (15.79) | 1 (1.59) | 0.006 |
| Unchanged | 7 (19.4) | 3 (9.38) | 0.314 | 10 (17.54) | 0 (0.00) | <0.001 |
| **Treatment Duration** |  |  |  |  |  |  |
| Total LOS |  |  |  |  |  |  |
| Pathogenicity |  |  |  |  |  |  |
| Colonization | 22.00 (16.00-30.00) | 38.50 (23.75-49.00) | 0.077 | 28.00 (15.75-51.00) | 38.50 (23.75-49.00) | 0.726 |
| Infection | 21.00 (12.50-71.00) | 49.00 (26.75-77.25) | 0.126 | 28.00 (16.50-57.00) | 22.50 (11.00-54.00) | 0.471 |
| Outcome |  |  |  |  |  |  |
| Recovery | 21.00 (15.00-34.00) | 43.00 (36.50-70.00) | 0.002 | 25.00 (14.50-43.00) | 24.00 (15.00-51.25) | 0.845 |
| Deterioration | 27.00 (12.50-75.25) | 25.50 (17.50-41.00) | 0.948 | 29.00 (25.50-97.50) | 10.00 (10.00-10.00) | 0.127 |
| Post-MDRO LOS |  |  |  |  |  |  |
| Pathogenicity |  |  |  |  |  |  |
| Colonization | 10.00 (6.50-16.00) | 18.00 (10.50-33.25) | 0.085 | 12.00 (7.00-26.00) | 9.50 (7.00-27.00) | 0.785 |
| Infection | 9.00 (5.50-38.50) | 30.00 (18.00-51.25) | 0.086 | 15.50 (9.50-30.25) | 14.00 (6.00-37.50) | 0.518 |
| Outcome |  |  |  |  |  |  |
| Recovery | 11.00 (9.00-16.00) | 32.00 (15.50-47.00) | 0.005 | 12.00 (7.00-22.25) | 12.50 (7.00-27.00) | 0.915 |
| Deterioration | 7.50 (4.75-22.50) | 16.50 (6.75-26.25) | 0.605 | 17.00 (15.00-62.50) | 6.00 (6.00-6.00) | 0.124 |
| Post-MDRO antibiotic course |  |  |  |  |  |  |
| Pathogenicity |  |  |  |  |  |  |
| Colonization | 9.00 (6.00-13.50) | 9.00 (6.00-25.00) | 0.676 | 6.50 (2.75-20.25) | 7.00 (5.00-17.00) | 0.866 |
| Infection | 8.00 (5.00-28.50) | 17.00 (11.00-28.00) | 0.394 | 12.00 (6.00-18.00) | 10.00 (6.00-14.25) | 0.528 |
| Outcome |  |  |  |  |  |  |
| Recovery | 8.50 (6.50-16.50) | 17.00 (6.75-28.00) | 0.262 | 7.00 (4.00-17.50) | 8.00 (5.00-14.00) | 0.607 |
| Deterioration | 7.50 (4.50-21.25) | 16.50 (6.75-26.25) | 0.475 | 17.00 (10.00-27.00) | 6.00 (6.00-6.00) | 0.14 |
|  |  |  |  |  |  |  |
|  |  |  |  |  |  |  |
| **Antibiotic consumption** |  |  |  |  |  |  |
| Total hospital stay |  |  |  |  |  |  |
| Pathogenicity |  |  |  |  |  |  |
| Colonization | 38.59 (21.18-49.07) | 42.82 (29.33-79.46) | 0.454 | 25.66 (7.54-41.52) | 15.75 (8.85-33.33) | 0.591 |
| Infection | 26.50 (18.77-53.36) | 79.13 (30.33-139.84) | 0.031 | 23.59 (11.89-67.09) | 19.58 (11.34-44.63) | 0.585 |
| Outcome |  |  |  |  |  |  |
| Recovery | 36.50 (18.00-49.27) | 62.33 (31.09-108.63) | 0.015 | 20.44 (9.00-31.66) | 17.20 (9.59-39.50) | 0.964 |
| Deterioration or death | 36.63 (19.43-79.77) | 79.44 (35.18-132.39) | 0.439 | 59.26 (32.84-72.25) | 20.41 (20.41-20.41) | 0.275 |
| After MDRO positive |  |  |  |  |  |  |
| Pathogenicity |  |  |  |  |  |  |
| Colonization | 11.79 (1.94-26.16) | 12.41 (0.84-37.5) | 0.647 | 3.75 (1.05-14.82) | 5.66 (0.86-11.28) | 0.969 |
| Infection | 10.04 (5.64-25.76) | 57.40 (17.42-85.56) | 0.021 | 17.15 (2.30-32.12) | 10.60 (6.38-38.13) | 0.524 |
| Outcome |  |  |  |  |  |  |
| Recovery | 10.04 (1.77-28.13) | 37.00 (7.50-66.14) | 0.019 | 4.25 (1.78-17.36) | 8.06 (3.83-21.45) | 0.247 |
| Deterioration or death | 15.13 (4.75-23.15) | 51.27 (10.39-85.30) | 0.156 | 15.20 (4.40-42.13) | 11.20 (11.20-11.20) | 0.827 |
| **Total antibiotic cost ($)** |  |  |  |  |  |  |
| Pathogenicity |  |  |  |  |  |  |
| Colonization | 1564.05 (879.72-2428.6) | 2311.8 (1381.17-3245.93) | 0.262 | 1257.04 (552.48-3630.65) | 864.54 (313.97-2658.98) | 0.107 |
| Infection | 2735.10 (913.19-3135.53) | 2728.14 (1945.17-6590.30) | 0.236 | 1394.87 (479.98-3306.75) | 706 (279.13-1832.75) | 0.293 |
| Outcome |  |  |  |  |  |  |
| Recovery | 2172.75 (745.12-2828.46) | 2601.73 (1920.51-4006.62) | 0.072 | 1116.53 (459.77-2449.49) | 738.02 (279.66-1886.87) | 0.273 |
| Deterioration or death | 2327.40 (1287.24-6310.56) | 4721.65 (1584.45-10022.28) | 0.439 | 3306.33 (2491.61-3570.36) | 2115.66 (2115.66-2115.66) | 0.275 |

Data are presented as median and interquartile range unless specified otherwise.

The cost was at the exchange rate as US $100 is approximately equal to 636.53 Chinese Renminbi (Date of conversion 9 April 2022).

Abbreviations: PEAC, pharmacist early initiative consultation; NEWS 2, National Early Warning Score 2; LOS, length of stay; MDRO, multi-drug resistant organisms.

^a^Disease Severity, the severity of the disease was distinguished by the NEWS 2 score on admission, with a score greater than or equal to 7 as severe, and less than 7 as mild to moderate.

**Supplementary Table 2. Efficacy Evaluation of MDRO-Infected Patients^a^ between Different Disease Severity^b^ at Admission**

|  | NEWS 2 ≥7 | | | NEWS 2 <7 | | |
| --- | --- | --- | --- | --- | --- | --- |
|  | non-PEAC  N=19 | PEAC  N=18 | *P-*Value | non-PEAC  N=24 | PEAC  N=32 | *P-*Value |
| **Infection treatment effect (n, %)** |  |  |  |  |  |  |
| Improvement | 8 (42.11) | 11 (61.11) | 0.476 | 15 (62.50) | 31 (96.88) | 0.002 |
| Deterioration | 6 (31.58) | 6 (31.33) | >0.999 | 4 (16.67) | 1 (3.13) | 0.074 |
| Unchanged | 5 (26.32) | 1 (5.56) | 0.142 | 5 (20.83) | 0 (0.00) | 0.020 |
| **Treatment Duration** |  |  |  |  |  |  |
| Total LOS |  |  |  |  |  |  |
| Recovery | 24.50 (14.25-62.00) | 74.00 (47.50-80.50) | 0.047 | 28.00 (16.00-58.00) | 25.00 (11.00-54.00) | 0.639 |
| Deterioration or death | 17.00 (11.50,54.00) | 25.50 (17.50-41.00) | 0.575 | 26.50 (20.75-45.75) | 10.00 (10.00-10.00) | 0.157 |
| Post-MDRO LOS |  |  |  |  |  |  |
| Recovery | 10.50 (9.00-37.25) | 39.00 (29.00-65.50) | 0.039 | 15.00 (10.50-27.00) | 14.00 (6.50-38.00) | 0.639 |
| Deterioration or death | 6.50 (4.25-8.75) | 16.50 (6.75-26.25) | 0.335 | 15.00 (11.75-22.00) | 6.00 (6.00-6.00) | 0.157 |
| Post-MDRO antibiotic course |  |  |  |  |  |  |
| Recovery | 9.00 (7.00-24.75) | 18.00 (14.00-42.75) | 0.363 | 9.00 (6.00-16.00) | 10.00 (6.00-14.50) | 0.934 |
| Deterioration or death | 6.50 (3.50-8.75) | 16.50 (6.75-26.25) | 0.294 | 14.50 (11.00-22.00) | 6.00 (6.00-6.00) | 0.060 |
| Starting improved days | 4.50 (3.25-10.25) | 5.00 (2.00-8.50) | >0.999 | 5.00 (4.00-6.50) | 3.00 (2.00-5.50) | 0.096 |
| **Antibiotic consumption** |  |  |  |  |  |  |
| Total hospital stay |  |  |  |  |  |  |
| Recovery | 45.83 (19.15-61.59) | 92.30 (52.27-135.07) | 0.083 | 23.75 (16.94-65.75) | 18.75 (10.78-48.75) | 0.504 |
| Deterioration or death | 22.79 (15.11-42.94) | 79.44 (35.18-132.39) | 0.150 | 50.96 (20.74-81.65) | 20.41 (20.41-20.41) | 0.480 |
| After MDRO positive |  |  |  |  |  |  |
| Recovery | 11.02 (4.03-33.32) | 61.80 (29.01-90.31) | 0.039 | 19.25 (2.20-28.23) | 10.00 (6.25-38.75) | 0.574 |
| Deterioration or death | 8.63 (2.25-17.06) | 51.27 (10.39-85.30) | 0.150 | 20.33 (6.54-38.50) | 11.20 (11.20-11.20) | >0.999 |
| **Total antibiotic cost ($)** |  |  |  |  |  |  |
| Recovery | 2781.78 (951.23-2892.59) | 2786.02 (2130.05-5729.53) | 0.215 | 782.59 (463.24-1717.44) | 665.69 (262.29-1584.44) | 0.665 |
| Deterioration or death | 2327.4 (998.01-5368.97) | 4721.65 (1584.45-10022.28) | 0.337 | 2985.11 (2198.99-3402.11) | 2115.66 (2115.66-2115.66) | 0.480 |

Data are presented as median and interquartile range unless specified otherwise.

The cost was at the exchange rate as US $100 is approximately equal to 636.53 Chinese Renminbi (Date of conversion 9 April 2022).

Abbreviations: PEAC, pharmacist early initiative consultation; NEWS 2, National Early Warning Score 2; LOS, length of stay; MDRO, multi-drug resistant organisms.

^a^MDRO-Infected Patients, the detected pathogen is considered to be causing the infection in the patient.

^b^Disease Severity, the severity of the disease was distinguished by the NEWS 2 score on admission, with a score greater than or equal to 7 as severe, and less than 7 as mild to moderate.
